# Amelanotic nodular melanoma misdiagnosed as a benign skin lesion: A rare case report from Syria

**DOI:** 10.1016/j.amsu.2022.103316

**Published:** 2022-01-26

**Authors:** Mohammed Moutaz Alshaghel, Lana Almahairi, Roua Arian, Mohamad Shehab Alyousfi, Weaam Majanni, Rama Alyousfi, Aladdin Etr

**Affiliations:** a Faculty of Medicine, Aleppo University, Aleppo, Syria; b Department of Dermatology, Aleppo University Hospital, Aleppo University, Aleppo, Syria; c Department of Pathology, Aleppo University Hospital, Aleppo University, Aleppo, Syria; d Department of Plastic Surgery, Aleppo University Hospital, Aleppo University, Aleppo, Syria

**Keywords:** Melanoma, Amelanotic, Nodular, Heel, Surgical procedure

## Abstract

**Introduction:**

and importance: Amelanotic melanoma is a rare and aggressive type of melanoma. It is often diagnosed late because of the lack of melanin in its cells, and this causes treatment delay and, eventually, poor prognosis.

**Case presentation:**

We report a case of a 79-year-old female patient that presented to the dermatology clinic with an asymptomatic lesion on the medial heel of the right foot, with no medical history of previous melanoma or related skin cancer. To get the right diagnosis, an incisional biopsy was performed, and the sample was sent to the pathology laboratory. The sample was stained with S100 and HMB-45 stains, and both were positive. Also, no melanin pigmented cells were seen, so the diagnosis was amelanotic nodular melanoma. The patient was then referred to surgery. The lesion was successfully excised with 5cm safety margins, and the whole lesion was sent to the pathology laboratory to ensure that the edges are malignancy-free. After 18 months of follow-up, the patient is in good health.

**Conclusion:**

Accurate and early diagnosis with appropriate clinical intervention can improve the prognosis and reduce mortality and morbidity rates.

## Highlights

• Amelanotic melanoma is typically misdiagnosed with other skin lesions with similar characteristics, especially those on the foot.
• Accurate diagnosis is often delayed, resulting in a poor prognosis which makes AM a challenging case.
• Early and accurate diagnosis of the lesion using histological and immunohistochemical examinations can improve the prognosis and maintain the patient's quality of life.
• Surgical excision of the lesion with appropriate margins of safety is the current approved treatment while other treatments are still being researched.

## 1 Introduction

Amelanotic melanoma (AM) is an extremely uncommon form of cutaneous melanoma and could be a subtype of all types of melanoma [1,2]. Patients diagnosed with AM are more arguably to have a challenging case due to the absence of objective diagnostic criteria. In addition, AM comprises only 8% of all melanoma cases, and this is the importance of our case [3,4]. The absence of melanin pigmentation clinically or dermoscopicaly (although some melanin granules could be seen on histopathological examination) is another reason for the misdiagnosis, and this can lead to the fact that the actual number of the disease cases is more than the reported confirmed number [[1], [5], [6]]. AM could be detected in unusual areas like the foot, which makes the lesion even more challenging to be detected due to other underlining skin lesions, such as warts, infections, ulcers, foreign body, and even psoriasis [7]. Here we report a rare case of a nodular acral amelanotic melanoma.

This case report has been reported in line with the SCARE Criteria [8].

## 2 Case Presentation

**Fig. 1:**
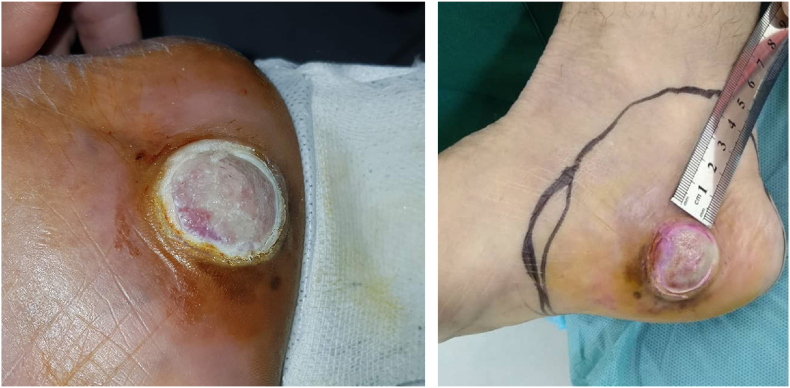
Clinical photograph showing asymptomatic mass with surgical safety margins.

A 79-year-old female presented to the dermatology clinic with an asymptomatic progressive lesion over the medial heel of the right foot from a year ago [Fig. 1].

The patient was seen by a local general doctor in her village, and treated by local ointments; however, the lesion continued to increase in size; which prompted her doctor to refer her to the central hospital.

Gross examination of the mass revealed a 3-cm-diameter lesion, with no ulceration or any other suspicious marks. The patient's medical history included hypertension treated by beta-blockers.

Her surgical history included an umbilical hernia 10 years ago. No family history of melanoma or other skin cancers has been reported.

There was apparent shortness of breath on exertion with no chest pain. Physical examination showed a supraumbilical painless lump. The patient's blood pressure was 130/60 mmHg, and her heart rate was 110 bpm.

Blood tests included CBC, glucose, electrolytes, creatinine, urea, ALT, AST, bilirubin, albumin, and cholesterol. All were within normal limits.

**Fig. 2:**
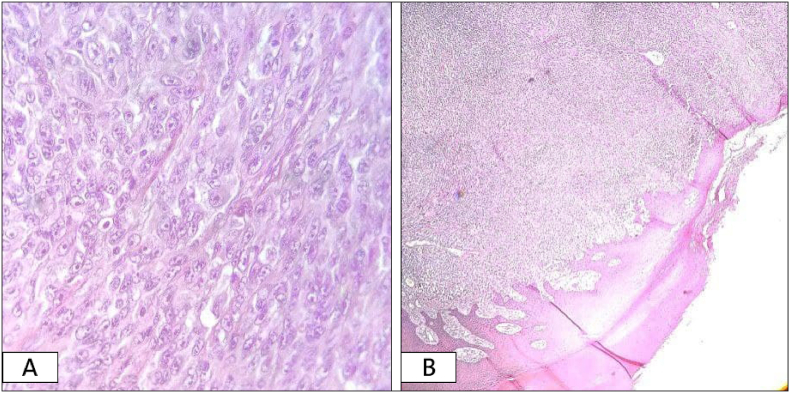
Sections show ulcerated epidermis, the dermis show infiltration of malignant melanocytes that are highly pleomorphic, presence of prominent nucleoli and atypical mitotic figures. (A) Hematoxylin & eosin, X40. (B) Hematoxylin & eosin, X4.

Several differential diagnoses were considered; therefore, a biopsy was suggested. Incisional biopsy of the lesion (Size3*2.5*1cm) was studied. Sections showed ulcerated epidermis, and malignant proliferation of anaplastic melanocytes arranged in small nests invading the dermis, these cells had large hyperchromatic pleomorphic nuclei, prominent nucleoli, and some atypical mitoses, and no melanin pigmented cells were seen [Fig. 2].

**Fig. 3:**
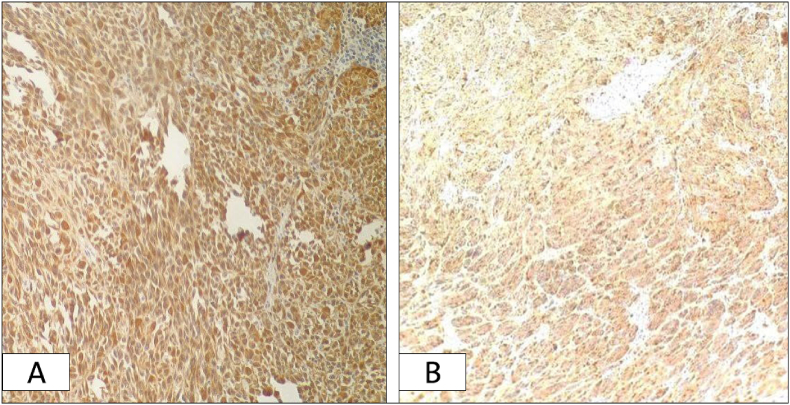
(A) Immunohistochemistry for S100 (nuclear stain), is positive in tumor cells. (B) Immunohistochemistry for HMB-45 (cytoplasmic stain), is positive also.

Immunohistochemically, the sections were stained with S100 and HBM-45, and both were positive [Fig. 3].

The diagnosis was confirmed as amelanotic melanoma (nodular type), and the patient was referred to a plastic surgeon to excise the lesion. CT scan of the abdomen, chest, and pelvic has been conducted and showed no metastasis.

**Fig. 4:**
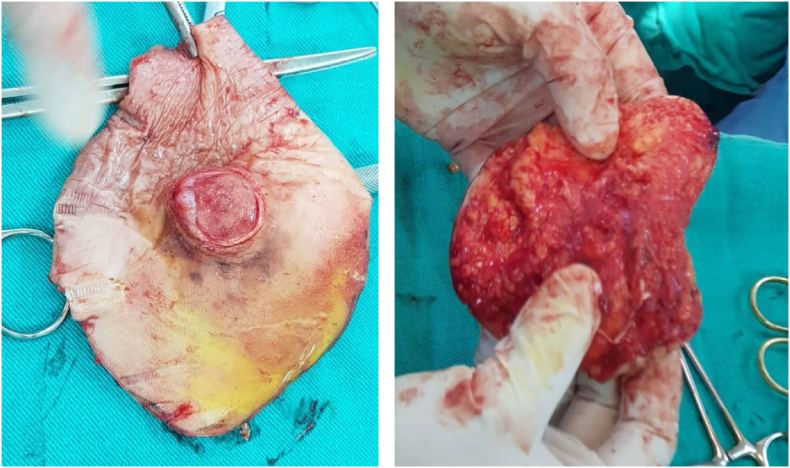
Surgical eradiation of the mass with 5 cm safety margins.

**Fig. 5:**
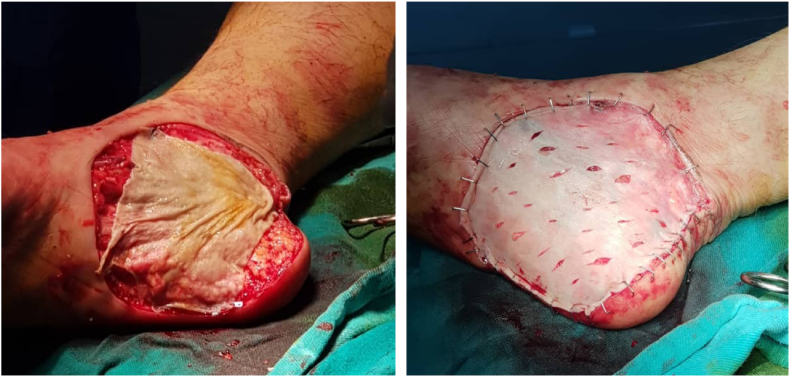
Fixation of tie-over beads with sterile bandages.

The surgery was performed under local anesthesia of the sciatic nerve. The lesion was excised with 5cm safety margins, and with a depth of 1.5 cm up to the muscle's aponeuroses [Fig. 4]. A partial-thickness skin graft was taken from the right thigh area, and tie-over beads were placed and fixed with a sterile bandage [Fig. 5]. Unfortunately, the sentinel node biopsy could not be performed due to limitations and lack of resources in the case's country.

An excisional biopsy was also performed and sent to the pathology lab (Size11 × 9.5 × 1.5cm). Sections showed malignant proliferation of anaplastic melanocytes dispersed through the dermis extending to the subcutaneous fat. The cells had large hyper-chromatic pleomorphic nuclei, prominent nucleoli, and eosinophilic cytoplasm with many scattered atypical mitoses.

Excisional biopsy showed a Breslow tumor thickness of at least 1.5 mm, Clark level IV (invasion of the reticular dermis), and surgical borders marked the absence of malignant cells. The lesion has been scraped, so the maximum tumor thickness could not be determined. However, we think that the Breslow depth is significantly greater than 1.5mm due to other prognostic factors.

**Fig. 6:**
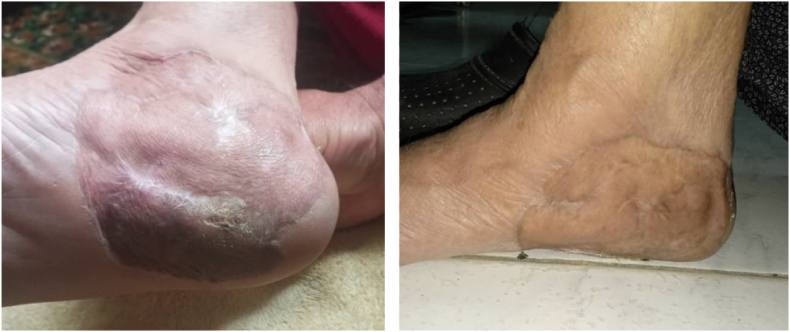
Surgical site after 4 months of surgery.

The follow-up after 4 months included physical examination, blood tests, and breast Echo. All findings were normal [Fig. 6]. The Follow-up lasted up to 18 months.

**Fig. 7:**
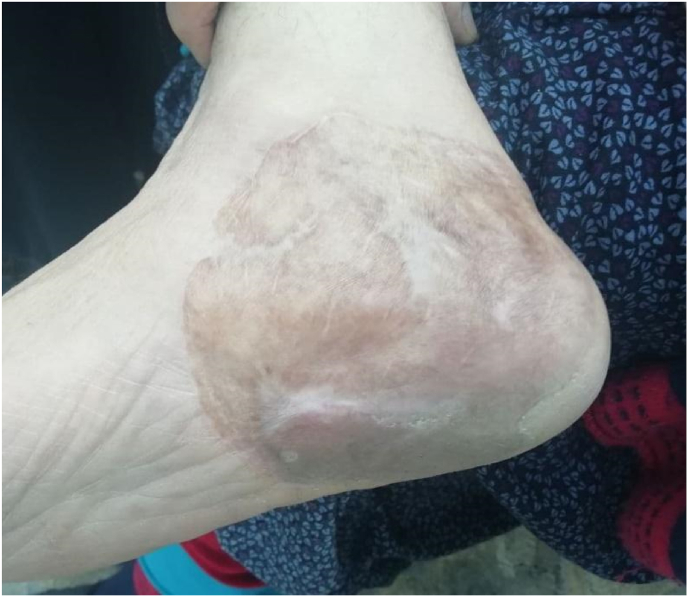
Surgical site after 18 months of surgery.

After 18 months of the follow-up, the patient was in good health [Fig. 7].

## 3 Discussion

Malignant Melanoma (MM) is a rare aggressive cancer, but the number of cases is increasing worldwide [9,10].

MM usually appears in the skin, but can present in any part of the body. Major types of cutaneous melanoma are superficial spreading melanoma (SSM), lentigo maligna melanoma (LMM), acral lentiginous melanoma (ALM), and nodular melanoma (NM) [10]. Amelanotic melanoma (AM) is a rare subtype of melanoma, identified by the lack of melanin pigment (hypomelanotic melanoma) or truly AM (non-pigmented) which is rarer.

Truly AM can be identified by the presence of melanin in less than 5% of tumor cells on histological examination. As seen in our case, no pigmentation at all appeared in the pathological examination [11,12]. AM accounts for 0.4–27.5% of melanoma cases in total. Moreover, AM can imitate different benign and malignant shapes of tumors, therefore it refers to any type of melanoma including the nodular type, as in our case [12]. However, non-pigmented nodular melanoma accounts for about 1% of all diagnosed melanomas [13]. AMs are most common among people older than 50 years with light skin color. In addition, having red hair, freckles, type I skin, sun-sensitive phenotype, and the previous history of AM could be risk factors [12]. The median thickness of amelanotic melanomas is 1.6 mm [11]. The thickness of the lesion in our case is above average with Clark level IV up to the reticular dermis. The fact that AMs may imitate many morphologies and appear in unusual areas explains why misdiagnosis rates can be as high as 89% [12]. When AMs appear in acral sites, it becomes more likely to confuse with other skin lesions such as diabetic foot ulcer, tinea, wart, pyogenic granuloma, basal cell carcinoma, vascular lesions, or benign nevi [[12], [13], [14]]. Delayed diagnosis can lead to increased mortality, morbidity, and, consequently, health care expenditures [13]. In our case, after the lesion became visible to the patient, her belief that the lesion was simple and treatable pushed her to delay seeking medical assessment for a whole year. In addition, AMs may have a faster growth rate than other pigmented melanomas [11].

AM does not display the classic characteristics of melanoma summarized by the acronym "ABCDE", which is used to distinguish cases that need a dermatologist. Bristow et al. established "CUBED", specific criteria based on the macroscopic features and clinical progression of the lesion. The patient should be referred to a specialist when two criteria are presented [13,15].

The golden diagnosis method is the histological examination in combination with immunohistochemistry stains, and since AM can show various cytological features, histological examination alone is insufficient. Histological examination shows cords or nests of atypical melanocytes found in the dermis [12].

S100 is the essential marker for melanoma. There are many other practical markers like Melan-A, HMB-45, tyrosinase, MITF, and Ki-67, which can be used to confirm the diagnosis. In some challenging cases, the electron microscope is used to obtain accurate results [12].

The prognosis depends on the histological thickness of the excised tumor (Breslow depth) and tends to be poor in patients with AM.

Several studies based on a large number of case series demonstrate a significant rate of mortality and tumor recurrences, as well as lower melanoma-specific survival (MSS), melanoma-free survival (MFS), 5-year survival, and overall survival (OS) [12,16].

Treatment of AM is the same as for pigmented melanoma; extensive excision of the lesion with appropriate safety margins. The estimation of safety margins is still controversial but depends on the lesion's development and the initial tumor's thickness.

Due to the results of several studies, safety margins of 2 cm are considered sufficient for tumors of 1–4 mm Breslow depth [6,[17], [18], [19]]. This method was applied in our case, and due to high Breslow thickness, the surgeon created additional 3 cm safety margins to ensure that the edges were free of malignancy [6,17]. Unfortunately, there are no adjuvant therapies available for this tumor, but clinical trials of adjuvant therapy are promising [17].

## 4 Conclusion

Amelanotic melanoma (AM) is often misdiagnosed, most commonly when the lesions are presented in the feet due to other relevant pathologies; therefore, other differential diagnoses are likely to be considered, especially since AM does not follow the standard melanoma progression. Early and accurate diagnosis can significantly improve the prognosis. Both histological and immunochemical examinations are required. Extensive eradication with appropriate safety margins is the best treatment.

## Declaration of competing interest

Authors declare that there is no conflict of interest.

## Ethical approval

No need.

## Sources of funding

None.

## Author contributions

- Aladdin Etr did the surgery.

- Weaam Majanni diagnosed the case in cooperation with pathology department.

- Rama Alyousfi made the histological and immunohistochemical examination.

- Mohammed Moutaz Alshaghel, Lana Almahairi, Roua Arian, and Mohamad Shehab Alyousfi wrote the manuscript.

## Informed consent

Written informed consent was obtained from the patient for publication of this case report and accompanying images. A copy of the written consent is available for review by the Editor-in-Chief of this journal on request.

## Registration of research studies

1. Name of the registry:
2. Unique Identifying number or registration ID:
3. Hyperlink to your specific registration (must be publicly accessible and will be checked):

## Guarantor

Mohammed Moutaz Alshaghel

m.moutaz.alshaghel@gmail.com.

## Provenance and peer review

Not commissioned, externally peer-reviewed.
